# Genome-wide methylation profiling of Beckwith-Wiedemann syndrome patients without molecular confirmation after routine diagnostics

**DOI:** 10.1186/s13148-019-0649-6

**Published:** 2019-03-21

**Authors:** I. M. Krzyzewska, M. Alders, S. M. Maas, J. Bliek, A. Venema, P. Henneman, F. I. Rezwan, K. v. d. Lip, A. N. Mul, D. J. G. Mackay, M. M. A. M. Mannens

**Affiliations:** 1 Amsterdam UMC, University of Amsterdam, Department of Clinical Genetics, Amsterdam Reproduction & Development, Meibergdreef 9, 1105 AZ Amsterdam, The Netherlands; 2 Amsterdam UMC, University of Amsterdam, Department of Pediatrics, Meibergdreef 9, 1105 AZ Amsterdam, The Netherlands; 3 Faculty of Medicine, University of Southampton, Southampton, UK

**Keywords:** BWS, MLID, DNA-methylation, Imprinting disorders

## Abstract

Beckwith-Wiedemann syndrome (BWS) is caused due to the disturbance of imprinted genes at chromosome 11p15. The molecular confirmation of this syndrome is possible in approximately 85% of the cases, whereas in the remaining 15% of the cases, the underlying defect remains unclear. The goal of our research was to identify new epigenetic loci related to BWS. We studied a group of 25 patients clinically diagnosed with BWS but without molecular conformation after DNA diagnostics and performed a whole genome methylation analysis using the HumanMethylation450 Array (Illumina).

We found hypermethylation throughout the methylome in two BWS patients. The hypermethylated sites in these patients overlapped and included both non-imprinted and imprinted regions. This finding was not previously described in any BWS-diagnosed patient.

Furthermore, one BWS patient exhibited aberrant methylation in four maternally methylated regions—*IGF1R*, *NHP2L1*, *L3MBTL*, and *ZDBF2*—that overlapped with the differentially methylated regions found in BWS patients with multi-locus imprinting disturbance (MLID). This finding suggests that the BWS phenotype can result from MLID without detectable methylation defects in the primarily disease-associated loci (11p15). Another patient manifested small but significant aberrant methylation in disease-associated loci at 11p near *H19*, possibly confirming the diagnosis in this patient.

**Electronic supplementary material:**

The online version of this article (10.1186/s13148-019-0649-6) contains supplementary material, which is available to authorized users.

## Introduction

Beckwith-Wiedemann syndrome (BWS) (OMIM 130650) is an overgrowth disorder with predisposition to embryonal tumor development. Its clinical symptoms include macrosomia, macroglossia, and abdominal wall defects, whereas minor features include ear pits, hypoglycemia, nephromegaly, and hemihypertrophy. BWS is clinically heterogeneous, and the major features may not manifest immediately after birth but appear in the first month of life [1].

**Fig. 1:**
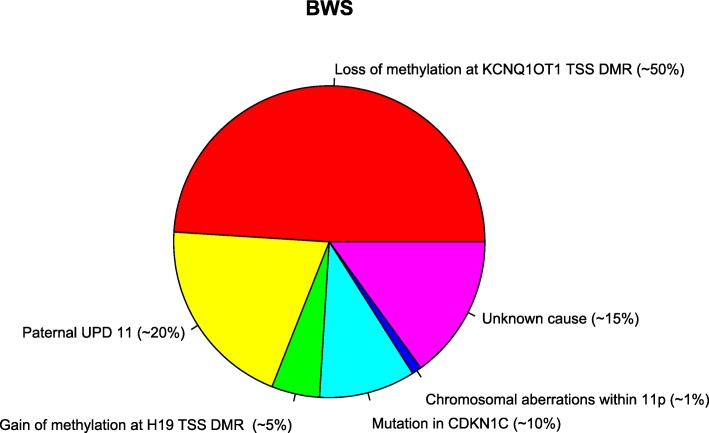
(Epi)genetic aberrations in BWS

Most cases (about 85%) of BWS result from various genetic and epigenetic aberrations in the imprinted region on chromosome 11p15.5. This region contains two imprinting centers: *H19* TSS DMR (transcription start site differentially methylated region) and *KCNQ1OT1* TSS DMR. The former regulates the transcription of genes *IGF2* and *H19*, whereas the latter regulates, among others, *KCNQ10T1* and *KCNQ1* [2] in a tissue-dependent manner. About 50% of these cases are caused by a loss of methylation at *KCNQ1OT1* TSS DMR on the maternal chromosome and 20% by paternal uniparental disomy (UPD) of 11p15.5. Up to 5% have a gain of methylation on the maternal chromosome at *H19* TSS DMR. Between 5% and 10% of the cases are caused by a mutation in the maternal copy of *CDKN1C*. Finally, some cases are caused by chromosomal aberrations on chromosome 11, for example, microdeletions (< 1%), translocations (< 1%), inversions (< 1%), or duplications (< 1%) within the imprinted region of 11p15 (2). Furthermore, hypomethylation in a large number of imprinting disorders (IDs) occurs not only at a single locus but also at other imprinted loci. This phenomenon is known as multi-locus imprinting disturbance (MLID) and can be seen in about 30% of BWS with hypomethylation at *KCNQ1OT1* [3]. In about 15% of BWS, no (epi)genetic cause can be found (Fig. 1) [4].

The aim of this study was to identify new BWS-related epigenetic loci in a group of patients with unknown (epi)genetic cause. We searched for regions with altered methylation in known imprinted genes in each patient and regions with altered methylation shared between patients in the whole methylome.

## Methods

DNA was isolated from whole blood samples using Gentra Chemicals (Qiagen).

### Patient population

#### BWS patients

Twenty-five BWS patients (BWS1–BWS25) were devoid of any methylation defect in the imprinted region on chromosome 11p15.5 and a mutation in *CDKN1C*, as determined in the genome diagnostics department of the Academic Medical Center (the Netherlands). The imprinting defect at 11p15.5 was excluded using high resolution melting analysis (HRMA) [5] and/or methylation-specific multiplex ligation-dependent probe amplification (MS-MLPA). For both the methods, the limit of detection was 10% of alteration of methylation in comparison to negative controls. Maternally inherited mutations in *CDKN1C* were excluded using Sanger sequencing (see Additional file 1).

All the patients fulfilled the BWS criteria of DeBaun, displaying at least two of the five most common BWS features, including macrosomia, macroglossia, abdominal wall defects, ear creases/pits, and hypoglycemia [6, 7].

#### Positive controls (MLID-BWS)

We used five DNA samples from MLID-BWS patients with known hypomethylation at *KCNQ1OT1* TSS DMR and other imprinted loci as a positive control group to determine the robustness of this test. Hypomethylation in these patients was previously determined and described by Bliek et al. in 2008 [8]. The additional hypomethylated loci in these patients were as follows:

- MLID-BWS1: *MEST*, *GNAS_AS1*;
- MLID-BWS2: *GNAS_AS1*;
- MLID-BWS3: *GNAS_AS1*, *DIRAS3*;
- MLID-BWS4: *MEST*; and
- MLID-BWS5: *GBR10A*, *GNAS_AS1*, *SNRPN*.

#### Control group

Twenty-six DNA samples from healthy anonymous individuals were used as a normal control group.

### HumanMethylation450 array

The HumanMethylation450 array (Illumina, San Diego, USA) was used to obtain genome-wide methylation levels. This array comprises 485,000 CpG sites throughout the genome and covers 99% of RefSeq genes and 96% of CpG islands. In order to reduce technical bias, patients and controls were randomly divided over the analysis. DNA was bisulfite converted using the EZ DNA methylation kit of ZYMO® and subsequently submitted for analysis on the HumanMethylation450 array, outsourced to GenomeScan, Leiden, The Netherlands (ISO/IEC 17025 approved). The quality of raw methylation data was assessed using the MethylAid script in R (*GenomeScan*’*s Guidelines for Successful Methylation Experiments Using the Illumina Infinium*® *HumanMethylation BeadChip*). All the samples met the quality criteria. To analyze our data, we used a statistical method for single-sample analysis described by Rezwan et al. [9] adapted to a MINFI package in R (based on the Crawford-Howell *t* test). Prior to this analysis, all probes known to involve polymorphic sites (minor allele frequency, MAF > 0.01), cross-hybridization probes, and probes located on sex chromosomes were removed from the dataset. The workflow and QC details are depicted in the Additional file 1. The 450 k data was normalized with the function preprocessFunnorm of MINFI [10].

Differentially methylated positions (DMP) with assigned adjusted *P* value for M-values smaller than 0.05 were considered as significant (adjusted according to the false discovery rate; FDR).

### Searching for DMRs at imprinted loci

Next, we determined whether significant DMPs were located in the imprinted regions. To correctly identify imprinted regions in our data, we used chromosomal locations for imprinted DMRs published by the European Network for Human Congenital Imprinting Disorder. These regions will be referred to as COST regions. (standardized nomenclature for imprinted loci/DMR; http://www.imprinting-disorders.eu/?page_id=3154). Note that COST regions in imprinted loci are differentially methylated alleles. (GenomicRanges version 1.28; R-software).

We calculated the percentage of significant DMPs that were located in COST regions for each patient. Further, to avoid false-positive results, only COST regions with two or more significant DMPs were considered as potential aberrantly methylated regions.

### Searching for new DMRs shared between BWS patients

To test whether significant DMPs occurred in two or more BWS patients, we carried out a comparative analysis: (1) first, we selected significant DMPs based on the adj.*P*value_M < 0.05 per patient, taking into account the direction of aberration (hypomethylated; hypermethylated); (2) then, we compared selected DMPs between patients and filtered out DMPs that occurred in more than one BWS patient; (3) the last filter step involved a selection of at least two significant DMPs within the region (based on the gene name and index number of CpG sites). MLID-BWS patients were included in this analysis.

The implementation of these filter steps allowed for the identification of DMPs showing recurrent altered methylation in BWS patients rather than the biggest effect of size and/or the smallest *P* value.

## Results

### HumanMethylation450 array

In order to test the ability of the HumanMehylation450 array to detect alterations of methylation in BWS, we tested five MLID-BWS patients with known epimutations. Significant DMPs were seen in all known aberrantly methylated regions *(KCNQ1OT1*, *DIRAS3*, *GNAS*-*AS1*, *MEST*, *GRB10*, and *SNRPN*), indicating that this platform can detect methylation defects in BWS. Moreover, by using this method, we identified hypomethylation in 18 other imprinted loci *(DIRAS3_Ex2*, *IGF1R*, *GNAS*-*XL*, *WRB*, *FAM50B*, *FANCC*, *GNAS*-*A/B*, *NHP2L1*, *ERLIN2*, *MAGEL2*, *MCTS2P*, *PPIEL*, *PEG10*, *RB1*, *NDN*, *SNRPN_5′DMR4*, *SNRPN_variant4*, and *L3MBTL1*) and hypermethylation in two imprinted loci (*ZDBF2* and *GNAS*-*NESP*), which were not known to be aberrantly methylated in these five MLID-BWS patients (Table 1).

**Table 1:** Imprinted loci in MLID BWS patients detected by HumanMethylation450 array (Illumina)

	MLID BWS1	MLID BWS2	MLID BWS3	MLID BWS4	MLID BWS5	Number of patients
Hypomethylated						
KCNQ1OT1	●	●	●	●	●	5
DIRAS3	○	●	○	–	○	4
DIRAS3_Ex2	○	○	○	–	○	4
GNAS-AS1	●	●	●	–	●	4
GNAS-XL	○	○	○	–	○	4
WRB	–	○	○	○	○	4
IGF1R	–	–	○	○	○	3
FAM50B	–	○	○	–	○	3
FANCC	–	○	○	–	○	3
GNAS-A/B	○	○	○	–	–	3
NHP2L1	–	○	○	–	○	3
MEST	●	–	–	●	–	2
ERLIN2	–	○	–	–	○	2
MAGEL2	○	–	–	–	○	2
MCTS2P	–	–	○	–	○	2
PPIEL	–	–	–	–	○	1
GRB10	–	–	–	–	●	1
PEG10	–	–	–	○	–	1
RB1	○	–	–	–	–	1
NDN	–	–	–	–	○	1
SNRPN_5′DMR4	–	–	–	–	○	1
SNRPN_variant4	–	–	–	–	○	1
SNRPN/SNURF	–	–	–	–	●	1
L3MBTL1	○	–	–	–	–	1
Hypermethylated						
ZDBF2	○	○	○	–	○	4
GNAS-NESP	–	–	○	–	–	1

● and ○ aberrant methylation detected by HumanMethylation array (Illumina)

● previously known to be hypomethylated

We performed a single-sample analysis for each patient in our BWS cohort. The number of significantly hypomethylated DMPs per patient ranged from 184 (BWS3) to 967 (BWS24) while that of significantly hypermethylated DMPs ranged from 148 (BWS16) to 12,931 (BWS24) (Table 2).

**Table 2:** Number and percentage of significant DMPs per patient in 450 K

Patient_ID	Number of hypermethylated DMPs	% hypermethylated DMPs in 450 K	Patient_ID	Number of hypomethylated DMPs	% hypomethylated DMPs in 450 K
*BWS24*	*12,931*	*3.02*	BWS24	967	0.23
*BWS25*	*8504*	*1.98*	MLID BWS2	882	0.21
MLID BWS2	1897	0.44	MLID BWS3	833	0.19
BWS7	1619	0.38	BWS25	642	0.15
MLID BWS4	1275	0.30	MLID BWS1	586	0.14
MLID BWS1	1082	0.25	MLID BWS5	535	0.12
MLID BWS3	1070	0.25	BWS10	522	0.12
BWS5	1039	0.24	BWS9	482	0.11
BWS10	995	0.23	MLID BWS4	467	0.11
BWS1	805	0.19	BWS11	433	0.10
BWS22	749	0.17	BWS7	338	0.08
BWS11	669	0.16	BWS18	337	0.08
BWS4	552	0.13	BWS2	332	0.08
BWS8	453	0.11	BWS17	327	0.08
BWS3	431	0.10	BWS6	318	0.07
BWS9	421	0.10	BWS22	306	0.07
BWS2	403	0.09	BWS19	302	0.07
MLID BWS5	322	0.08	BWS5	294	0.07
BWS6	303	0.07	BWS16	293	0.07
BWS15	299	0.07	BWS4	282	0.07
BWS19	236	0.06	BWS1	279	0.07
BWS17	218	0.05	BWS13	265	0.06
BWS21	215	0.05	BWS23	265	0.06
BWS13	214	0.05	BWS12	264	0.06
BWS23	211	0.05	BWS14	258	0.06
BWS12	210	0.05	BWS15	252	0.06
BWS18	201	0.05	BWS21	248	0.06
BWS14	185	0.04	BWS8	248	0.06
BWS20	174	0.04	BWS20	245	0.06
BWS16	148	0.03	BWS3	184	0.04

In two patients, BWS24 and BWS25, we found hypermethylation throughout the methylome. We identified almost 13,000 hypermethylated DMPs in BWS24, which is more than 3% of all the informative probes included in the analysis. In BWS25, hypermethylated DMPs, significantly, accounted almost for 2% of all the informative probes. The percentage of hypermethylated DMPs in other patients did not exceed 0.44%.

BWS24 and BWS25 also exhibited a slightly increased number of hypomethylated DMPs in comparison to other BWS patients but not different from MLID-BWS patients. Significantly, hypomethylated DMPs accounted for 0.23% in BWS24 and 0.15% in BWS25 of all the informative probes included in the analysis. In MLID-BWS patients, this percentage ranged from 0.11% to 0.21%.

**Fig. 2:**
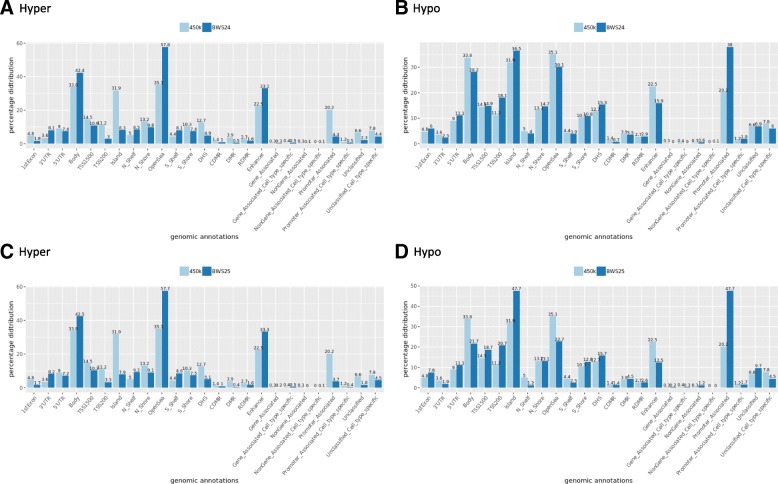
Genomic distribution of significant DMPs in patients BWS24 and BWS25. The plots compare the percentage of genomic distribution (genomic annotations of HumanMethylation450k array, Illumina) of all the informative probes included in our data and the percentage of genomic distribution of significant DMPs detected in patients BWS24 and BWS25. We compared the distribution of UCSC_RefGene_Group (1st exon, TSS200, TSS1500, 5′UTR, body, 3′UTR), Relation_to_UCSC_CpG_Island (Shore, Shelf, N, S), differentially methylated regions (DMR; CDMR, cancer-specific differentially methylated region; RDMR, reprogramming-specific differentially methylated region), overlapping enhancers loci, Regulatory_Feature_Group (Gene_Associated, Gene_Associated_Cell_type_specific, Promoter_Associated, Promoter_Associated_Cell_type_specific, Unclassified, Unclassified_Cell_type_specific), and DNase I hypersensitivity site (DHS). The number above each bar represents percentage. **a** The comparison of genomic distribution of hypermethylated DMPS in BWS24. **b** The comparison of genomic distribution of hypomethylated DMPs in BWS24. **c** The comparison of genomic distribution of hypermethylated DMPs in BWS25. **d** The comparison of hypomethylated DMPs in BWS25

The analysis of genomic distributions of significant DMPs of these two patients showed their similarity (Fig. 2). Hypermethylated significant DMPs were overrepresented in 3′ untranslated region (3′UTR), body, OpenSea, and enhancers while hypomethylated significant DMPs were overrepresented in Islands, DNase I hypersensitivity site (DHS), and Promoter_Associated.

To exclude the fact that these methylation patterns in these two patients are caused by cell type skewing, we estimated the distribution of cell types (function: estimateCellCounts, R package: FlowSorted.Blood.450k). Both the patients manifested comparable cell type profiles to the other patients in this group.

All significant DMPs per patient are listed in Additional file 2: Table S1 and Additional file 3: Table S2.

The estimation of the distribution of cell types and calculated *P* values are listed in Additional file 4: Table S3 and Additional file 5: Table S4.

### Searching for DMRs at imprinted loci

We calculated the percentage of total significant DMPs located in COST regions for each patient and for further analysis considered only COST regions with two or more significant DMPs as significantly aberrant (to avoid false-positive results).

MLID-BWS patients showed the highest percentage of significant DMPs assigned to COST regions, ranging from 3.73% to 17.97%, as expected (Table 3). In BWS24 and BWS25, the percentage of total significant DMPs located in COST regions was slightly high but lower than that in MLID-BWS patients. This percentage was 0.55% in BWS24 and 1.19% in BWS25. In contrast to MLID-BWS, BWS24 and BWS25 displayed hypermethylation in COST regions. However, the number of significant DMPs in these regions was relatively small, and the DMPs were non-consecutive. Both the patients displayed hypermethylation in *H19* TSS DMR (BWS24—8 DMPs, BWS25—26 DMPs), BWS24 additionally near *IGF2_*3′UTR (3 DMPs).

**Table 3:** Number and percentage of significant DMPs in COST DMRs

Patient_ID	Number of significant DMPs	Number of significant DMPs in COST DMR	% significant DMPs in COST DMPs
MLID BWS5	857	154	17.97
MLID BWS3	1903	226	11.88
MLID BWS1	1668	138	8.27
MLID BWS2	2779	161	5.79
MLID BWS4	1742	65	3.73
BWS7	1957	41	2.10
BWS22	1055	16	1.52
BWS25	9146	109	1.19
BWS5	1333	8	0.60
BWS24	13,898	77	0.55
BWS11	1102	6	0.54
BWS4	834	4	0.48
BWS23	476	2	0.42
BWS3	615	2	0.33
BWS1	1084	3	0.28
BWS16	441	1	0.23
BWS9	903	2	0.22
BWS12	474	1	0.21
BWS18	538	1	0.19
BWS17	545	1	0.18
BWS8	701	1	0.14
BWS10	1517	2	0.13
BWS13	479	0	0.00
BWS14	443	0	0.00
BWS15	551	0	0.00
BWS19	538	0	0.00
BWS20	419	0	0.00
BWS21	463	0	0.00
BWS2	735	0	0.00
BWS6	621	0	0.00

**Fig. 3:**
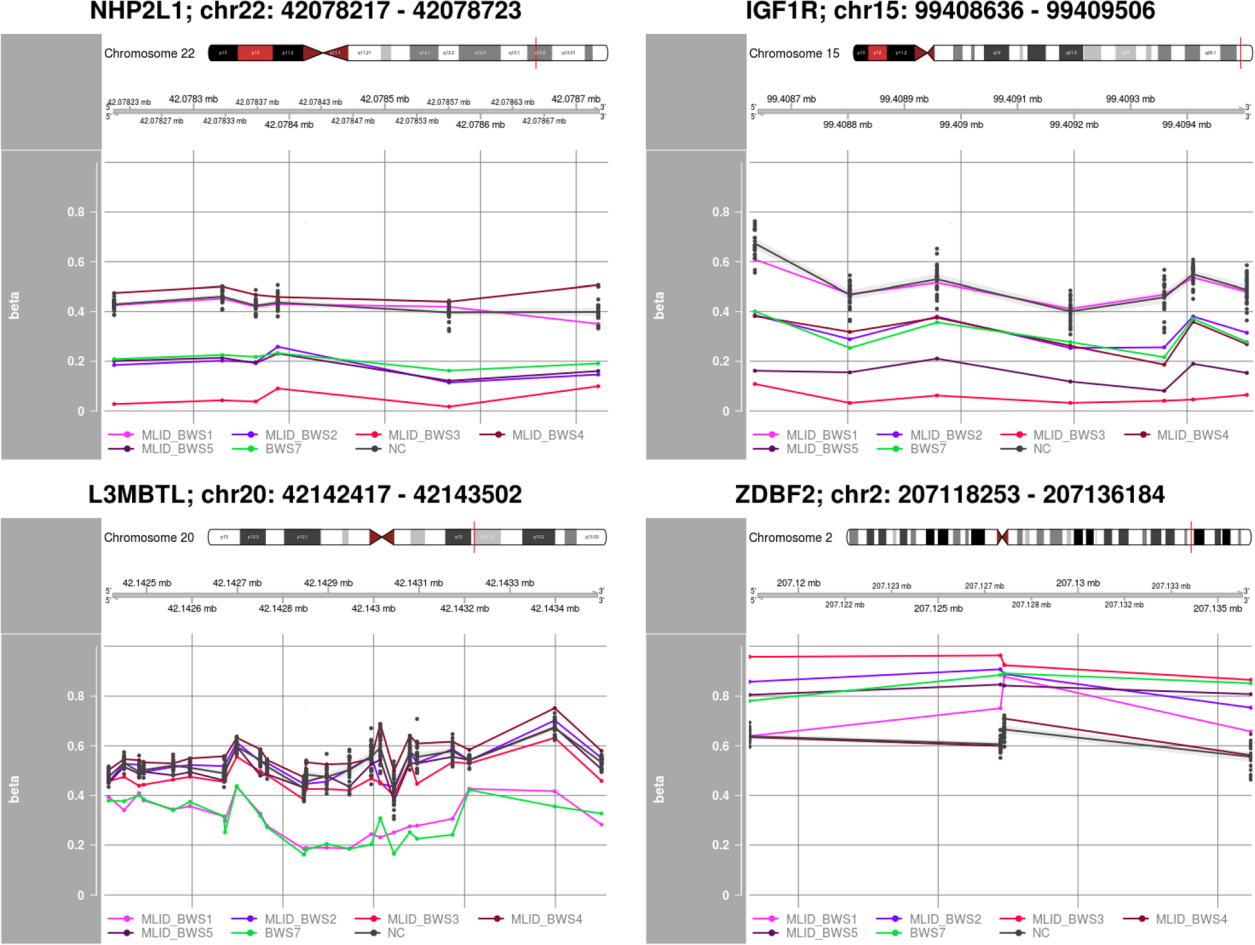
Aberrant methylated imprinted DMRs in BWS7 and MLID BWS patients. NC represents confidence interval of beta values in normal controls

In BWS7, we detected three hypomethylated DMRs (near *NHP2L1*, *IGF1R*, *and L3MBTL)* and one hypermethylated DMR (near *ZDBF2*) with two or more significant DMPs overlapping with COST regions in MLID-BWS patients (Fig. 3, Additional file 6: Table S5).

In BWS4, we detected alteration of methylation in the primary BWS-associated locus on 11p. In *KCNQ1OT1*, two consecutive hypomethylated DMPs were annotated with delta differences of − 0.37 and − 0.45. These two DMPs exhibited a stable methylation status in control with an adequate beta mean of 0.56 and 0.51 and standard deviation (SD) 0.03 and 0.04, respectively. Hypomethylation in this locus was not detected with standard diagnostics (see Additional file 1). Additional sequencing of this region (Sanger sequencing) showed the presence of SNP (rs190535862; minor allele frequency (MAF), *A* = 0.005; *G* > *A*) in one of the two hypomethylated CpGs positions in this patient, explaining the detected hypomethylation.

BWS22 displayed hypermethylation in *H19* TSS DMR in three nonconsecutive probes. The absolute delta difference in only one of these three DMPs was larger than 10%. However, all of them had a significant adj.P-value_M (all delta differences and adj.*P*-values assigned to each DMP per patient are listed in Additional file 1). The methylation status of all three DMPs in control was stable with SD 0.02, 0.01, and 0.02, respectively. Sanger sequencing did not show the presence of a SNP in this region. Moreover, we detected hypermethylation in this patient in two other imprinted loci assigned to *MEST* and *PEG13* (two significant DMPs in each).

The genomic locations of COST regions and the complete COST region analysis are listed in the Additional file 7: Table S6, Additional file 8: Table S7, and Additional file 9: Table S8.

### Searching for DMRs shared between BWS patients in the methylome

We compared significant DMPs between patients, including MLID-BWS patients.

**Fig. 4:**
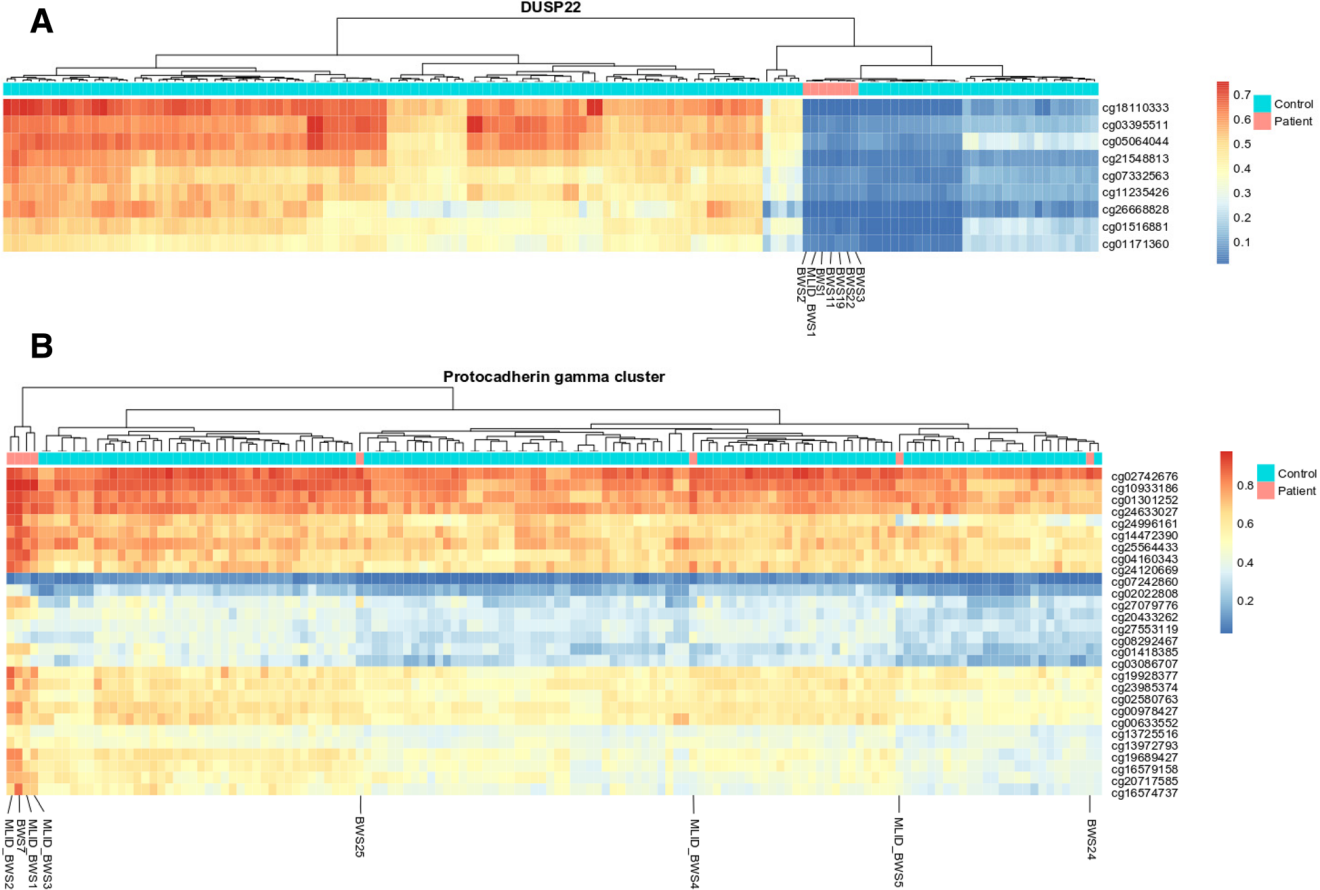
The heat maps of altered methylated probes from the comparative analysis. The blue beam represents healthy individuals from three different cohorts, and the red beam represents BWS patients. Normal controls come from three different batches that are visible on the plots. **a** Unsupervised hierarchical clustering of the beta values of the significantly hypomethylated probes annotated to the *DUSP22* detected in the current study. **b** Unsupervised hierarchical clustering of the beta values of the significantly hypermethylated probes annotated to the protocadherin gamma gene cluster detected in the current study

Regarding hypermethylation comparative analysis, we detected 6613 significant DMPs shared between a minimum of two BWS patients. A majority of them were shared between BWS24 and BWS25 in whom we detected hypermethylation throughout the methylome. We observed an increased number of hypermethylated DMPs annotated to the protocadherin gamma gene cluster located on chromosome 5. Thirty DMPs assigned to this cluster displayed hypermethylation in three MLID-BWS patients and BWS7. Further inspection of the 130 healthy individuals previously collected in our lab for diverse genome-wide methylation studies did not reveal any abnormal methylation in this region suggesting that alteration of methylation of this locus is specific for these patients (Table 4). Moreover, unsupervised hierarchical clustering of this region showed that the DNA pattern of BWS7 and three MLID-BWS patients fell in the same cluster (Fig. 4b).

**Table 4:** Most frequently altered methylated DMPs (the recurrent analysis)

Hypomethylated DMPs in DUSP22								
	MLID BWS1	BWS1	BWS2	BWS3	BWS11	BWS19	BWS22	Number of patients
cg07332563	○	○	○	○	○	○	○	7
cg21548813	○	○	○	○	○	○	○	7
cg03395511	○	○	○	○	○	○	○	7
cg15383120	○	○	○	○	○	○	○	7
cg18110333	○	○	○	○	○	○	○	7
cg05064044	○	○	○	○	○	○	○	7
cg11235426	○	○	○	○	○	○	○	7
cg01516881	○	○	○	○	○	○	○	7
cg26668828	○	○	○	○	○	○	○	7
cg01171360	○	○	○	○	○	○	○	7
Hypermethylated DMPs in protocadherin gamma gene cluster located on chromosome 5								
	MLID BWS1	MLID BWS2	MLID BWS3	MLID BWS4	BWS7	BWS24	BWS25	Number of patients
cg10933186	○	○	○		○			4
cg20717585	○	○	○		○			4
cg16574737	○	○	○		○			4
cg27079776	○	○			○		○	4
cg20433262	○	○	○		○			4
cg01418385	○	○			○			3
cg13725516	○	○			○			3
cg02580763	○	○			○			3
cg13972793	○	○			○			3
cg04160343	○	○			○			3
cg19928377		○	○		○			3
cg23985374	○	○						2
cg07242860	○				○			2
cg14472390	○				○			2
cg27553119	○	○						2
cg21537235		○		○				2
cg24120669		○			○			2
cg19689427		○			○			2
cg16579158		○			○			2
cg17760318		○			○			2
cg01301252		○			○			2
cg24996161		○			○			2
cg00978427		○			○			2
cg03086707		○			○			2
cg00633552		○			○			2
cg02022808		○			○			2
cg24633027		○			○			2
cg25564433		○			○			2
cg08292467		○		○				2
cg02742676						○	○	2

○ statistically significant aberrant methylation

We detected 1521 significantly hypomethylated DMPs shared between a minimum of two patients. More than 20% were annotated to imprinted loci (as reported above in the COST regions analysis) and shared between MLID-BWS patients. A majority of these shared probes represented single DMPs.

The most often altered methylated region was annotated to *DUSP22.* Ten DMPs located in this region shared hypomethylation between seven patients: MLID-BWS1, BWS1, BWS2, BWS3, BWS11, BWS19, and BWS22. The visualization of 130 healthy individuals showed variability in this specific region for healthy individuals (Fig. 4a), strongly suggesting that the hypomethylation of DMPs located near *DUSP22* detected in the current study is not specific for BWS.

The complete comparative analysis is listed in Additional file 10: Table S9.

## Discussion

We analyzed the methylation status of DNA in the cohort of 25 BWS patients devoid of any known (epi)genetic cause. We also included five MLID-BWS patients with known hypomethylated regions. Besides the known loci, we identified 18 other significantly hypomethylated and two hypermethylated imprinted regions. The percentage of significant DMPs in the COST regions was found to be higher in MLID-BWS than in other patients, indicating that the disruption of methylation is enriched in imprinted genes in MLID rather than genome-wide loss of methylation.

In patients BWS24 and BWS25, we detected hypermethylation throughout the methylome. BWS24 displayed hypermethylation in 3% and BWS25 in almost 2% of all the informative probes. This percentage is very high compared to that of other patients in whom hypermethylated DMPs did not exceed 0.44%. Both these patients also displayed hypermethylation in imprinted loci among others in *H19* TSS and additionally in *IGF2*_3′UTR in BWS24. However, the changes in methylation in these regions are subtle and do not clearly explain the development of BWS phenotype in these patients. The number of significant DMPs located in the COST regions was lower than that found in MLID-BWS patients, indicating aberrant methylation is not specific for imprinted regions, which suggests a different underlying mechanism to that in MLID patients. It has been shown recently that mutations in chromatin modifying genes cause aberrant methylation patterns throughout the genome. Possibly the methylation profile in our patients is caused by a mutation in such a gene [11].

In patient BWS7, we detected several hypomethylated imprinted loci that overlapped with MLID-BWS patients (*NHP2L1*, *IGF1R*, *L3MBT*L, and *ZDBF2*). All detected regions were previously described not only in MLID-BWS patients but also in other imprinting disorders showing MLID. For example, Docherty et al. described hypomethylation in the *NHP2LI* locus in Silver-Russell syndrome with MLID [12], and Rochtus et al. described hypomethylation in the *IGF1R* locus in patients with pseudohypoparathyroidism [13].

The most interesting of these four loci seems to be a locus situated near *IGF1R*. *IGF1R* is a receptor for *IGF1* and *IGF2*. *IGF2* is overexpressed in BWS cases with a gain of methylation at *H19* TSS DMR. *IGF1R* is normally a biallelic expression. However, Howard et al. reported a BWS case with monoallelic expression of the maternal *IGF1R* in a normal kidney, in Wilms tumor, and in lymphocytes. It is worth mentioning here that BWS patients with the gain of methylation in *H19* TSS DMR have a risk of Wilms tumor development [14]. In addition, many publications demonstrate that *IGF1R* affects birth weight and postnatal growth. This applies to both the overgrowth and the growth retardation. Okubo et al. reported two children with an abnormal growth were related to *IGF1R*. The first child with a growth retardation and hypoglycemia had only one copy of *IGF1R* while the second with overgrowth showed three copies of *IGF1R*. This suggests that loss of expression of *IGF1R* leads to growth retardation and over expression leads to overgrowth [15]. In BWS7, MLID-BWS3, MLID-BWS4, and MLID-BWS5, we detected a significant hypomethylation in the locus near *IGF1R*. The major feature of BWS is overgrowth, and we can speculate that hypomethylation of the *IGF1R* locus may lead to the over-expression of this gene and cause overgrowth in our patients. However, additional studies are necessary to ascertain this causal relationship.

In BWS4, we observed hypomethylation in two consecutive DMPs located in imprinted locus *KCNQ1OT1*, primarily associated with BWS. A SNP was present at the hybridization site of the probes of both CpGs. Knowledge of the fact that the existence of a SNP in the examined CpG can have an impact on methylation readouts [16], we believe that hypomethylation of the other DMP is a result of the cross-reaction between the two probes. This led to the conclusion that this finding is probably not the cause of BWS in this patient, and one should consider the presence of a SNP when only one or two nearby CpGs show hypomethylation.

BWS22 displayed gain of methylation in three nonconsecutive DMPs in the imprinted *H19* locus, and only one DMP had a delta difference larger than 10%. Although significant, it is not clear whether such a small number of aberrant DMPs can be causative for BWS. It may be that, due to tissue mosaicism, other tissues in this patient are more affected and aberrant methylation covers a larger region. Alders et al. reported three BWS patients with epigenetic changes in tongue and buccal swab but with normal methylation status in the blood [17]. The tissue mosaicism may also be a cause of BWS in other patients in our cohort.

We observed enriched hypermethylation in 30 DMPs annotated to the protocadherin gamma gene cluster located on chromosome 5 in three of five MLID-BWS patients and BWS7. The additional visualization of methylation patterns in healthy individuals showed that hypermethylation in this locus was specific for these three patients. Interestingly, BWS7 showed disturbed methylation in imprinted regions overlapping with MLID-BWS cases as well. This strengthens our hypothesis that BWS7 has MLID and led us to the conclusion that MLID patients display similar methylation patterns not only in imprinted but also in non-imprinted regions.

One region near the *DUSP22* gene showed profound hypomethylation in seven BWS patients, involving ten DMPs. A similar hypomethylation pattern was observed in 27 of 130 healthy individuals, indicating hypomethylation in this locus is not specific for BWS. The differential methylation in this gene has been so far reported in several methylation studies [18–20]. We observed aberrant methylation in this gene in other cohorts (data not published) as well. Steegenga et al. described changes in methylation in *DUSP22* in an age-related study and showed that changes in methylation in this gene do not influence gene expression and aberrant methylation may be due to interindividual variation [21]. Our results are in agreement with this hypothesis.

In summary, in our cohort of 25 BWS patients without a molecular conformation, we identified one patient with MLID, two patients with genome-wide hypermethylation, and one patient with only very subtle hypermethylation at the H19 locus. The results described here suggest that the HumanMethylation array is highly sensitive and can even detect a small alteration of methylation, which is helpful in confirming the diagnosis.

## Conclusions

BWS diagnosis cannot always be confirmed by routine diagnostic tests.

The results of our study indicate that the BWS phenotype may result from different epi(genetic) aberrations, and these aberrations do not necessarily have to be in the primarily disease-associated locus on 11p15. Additionally, we showed that the HumanMethylation450 array may be used to extend BWS diagnostics. This study points to unknown epi(genetic) mechanisms and paves the way for development of new diagnostics tests for BWS.

### Limitations

Our study was limited to lymphocytes, and we were not able to determine the methylation status in the imprinted region on chromosome 11p in other tissues and thereby excluded tissue mosaicism.

Moreover, the single case method does not include a correction for blood cell distribution, which may influence the results.

## Additional files

Additional file 1: Contains additional information about (1) quality control, (2) pre-processing data and statistical methods, (3) estimation of the cell type distribution, (4) standard diagnostics, and (5) additional inspection of primary BWS associated loci at 11p15.5. (DOCX 555 kb)

Additional file 2: **Table S1.** Significant hypermethylated DMPs per patient. (XLSX 3887 kb)

Additional file 3: **Table S2.** Significant hypomethylated DMPs per patient. (XLSX 1129 kb)

Additional file 4: **Table S3.** The estimation of the cell types distribution. (CSV 4 kb)

Additional file 5: **Table S4.** The calculation of *P* values of the cell types distribution per patient. (XLSX 47 kb)

Additional file 6: **Table S5.** The summary of identified aberrant CpGs within COST regions. (DOCX 25 kb)

Additional file 7: **Table S6.** Genomic locations of imprinted loci—COST regions. (CSV 2 kb)

Additional file 8: **Table S7.** Significant hypermethylated DMPs within the COST regions (per patient). BWS patients that did not display aberrant methylation in imprinted COST regions are not included in this table. (XLSX 38 kb)

Additional file 9: **Table S8.** Significant hypomethylated DMPs overlapped with COST regions. BWS patients that did not display aberrant methylation in imprinted COST regions are not included in this table. (XLSX 98 kb)

Additional file 10: **Table S9.** Results of the comparative analysis. (XLSX 5326 kb)

## Abbreviations

BWS: Beckwith-Wiedemann syndrome
DHS: DNase I hypersensitivity site
DMP: Differentially methylated position
DMR: Differentially methylated region
FDR: False discovery rate
HRMA: High resolution melting analysis
IDs: Imprinting disorders
MAF: Minor allele frequency
MLID: Multi-locus imprinting disturbance
MS-MLPA: Methylation-specific multiplex ligation-dependent probe amplification
QC: Quality control
SNP: Single nucleotide polymorphism
TSS: Transcription start site
