# Supplementary material for: Genome-wide methylation profiling of Beckwith-Wiedemann syndrome patients without molecular confirmation after routine diagnostics
Source: Clin Epigenetics. 2019 Mar 21;11:53. doi: 10.1186/s13148-019-0649-6 (PMC6429826; doi:10.1186/s13148-019-0649-6)
Supplement: Supplementary file 1 — Contains additional information about (1) quality control, (2) pre-processing data and statistical methods, (3) estimation of the cell type distribution, (4) standard diagnostics, and (5) additional inspection of primary BWS associated loci at 11p15.5. (DOCX 555 kb) [file 13148_2019_649_MOESM1_ESM.docx]

**Additional information**

**Genome-wide methylation profiling of Beckwith Wiedemann syndrome patients without molecular confirmation after routine diagnostics**

I.M. Krzyzewska^1*^, M.Alders^1*^, S. M. Maas^2^, J. Bliek^1^, A.Venema^1^, P. Henneman^1^, F.I. Rezwan^3^, K v. d. Lip^1^, A.N. Mul^1^, D. Mackay^2^ and M.M.A.M. Mannens^1#^

I.M. Krzyzewska^1*^, M.Alders^1*^, S. M. Maas^2^, J. Bliek^1^, A.Venema^1^, P. Henneman^1^, F.I. Rezwan^3^, K v. d. Lip^1^, A.N. Mul^1^, D.J.G. Mackay^3^ and M.M.A.M. Mannens^1#^

1 Amsterdam UMC, University of Amsterdam, Department of Clinical Genetics, Genome Diagnostics laboratory, Meibergdreef 9, Amsterdam, Netherlands; 2 Amsterdam UMC, University of Amsterdam, Department of Pediatrics, Meibergdreef 9, Amsterdam, The Netherlands; 3 Faculty of Medicine, University of Southampton, Southampton, UK

**Supplemental methods - flow scheme**

*Quality control:* DNA was isolated from whole blood samples using Gentra Chemicals (Qiagen). Randomized samples were sent to GenomeScan in Leiden (ISO/IEC 17025approved). A genome scan carried out the entry quality control, bisulfite treatment, quality control of bisulfite, hybridization of the HumanMethylation BeadChip, and Data QC according to the standardized protocol. QC reports were generated and all samples met the set quality criteria. In addition, we performed our own visualization of raw data using the MethylAid package (version 1.10.0) available for R-software that detects bad-quality samples (outliers). We used default thresholds for 450K: MU = 10.5, NP = 11.75, BS = 12.75, HC = 13.25, and DP = 0.95 = 0.95 (MU: the median methylated and unmethylated log 2 intensity plot; NP: overall sample-depend control plot; BS: bisulfite conversion quality control plot; HC: hybridization quality plot; DP: detection p-value plot).

Visualization of the raw data is shown in Figure 1. Next, we performed a PCA analysis of the raw data, in which cross-hybridization and XY probes were removed. Figure 2 shows the second principal component analysis. Furthermore, we evaluated the overall distribution of beta values per patient. Figure 3 shows the density plots of one patient (MLID-BWS1) as an example. All density plots for each patient are available on request.

*Pre-processing and statistical test:* After data quality assessment, the image files were loaded onto the R-software (3.4.3) with the MINFI (1.24.0) function read.metharray.exp. The data was normalized using the preprocessFunnorm function of MINFI. Probes with a SNP in the CPG with a MAF higher than 0.01 as reported on the SBE (the single base extension) list as well as cross-hybridization probes were removed with the dropLociWithSnps function in R. To avoid any gender bias within the samples, probes located on X and Y chromosomes were removed. All cases and controls were drawn from the same batch; therefore, a batch correction was not needed. Data was analyzed using a statistical method for the single sample analysis of HumanMethylation450 based on the Crawford-Howell t-test (CH t-test), as described by F.Rezwan et al. (2015)[1]. Beta-values (the percentage of methylation for each DMP) were converted to logit transformed M-values. P-values were determined for M-values with CH t-test. To minimize false positive results, we carried out the FDR adjustment (adj.P.value_M). DMPs with adj.P.value_M less than 0.05 were considered significant. Figure 4 depicts the workflow of the single-case analysis of this study.

**Estimation of the distribution of the cell type**

Since it is known that cell type skewing may influence the DNA methylation profile, we estimated the white blood cell distribution in each sample using “estimateCellCounts” function in “FlowSorted.Blood.450k” R-package. These counts were estimated for CD8T (Cytotoxische T-cell ), CD4T(T helper cells), NK (Natural killer cells), Bcell (B lymphocytes), Mono (Monocytes), and Gran (Granulocytes). For each cell type, P-values were calculated based on the Crawford-Howell t-test (Bonferroni correction). We did not observe any cell type skewing in our data. Only one exception was seen in the case of increased number of NK cells in BWS18 (0,18); however, this patient did not show any specific methylation pattern (Additional tables 3 and 4).

**Standard diagnostics**

DNA samples of 25 BWS patients were isolated from whole blood samples (Gentra Chemicals; Qiagen). A BWS diagnostic test was performed in the Genome diagnostics laboratory of the Amsterdam UMC (location AMC, The Netherlands). To detect imprinting defects in primarily BWS associated regions, two different tests were used: high resolution melting analysis (HRMA) and methylation-specific multiplex ligation-dependent probe amplification (MS-MLPA).

*High Resolution Melting Analysis*

Bisulfite-treated (EZ DNA Methylation™ kit, Zymo Research) targeted DNA of 25 BWS patients was amplified through PCR and analyzed directly using high resolution melting analysis (HRMA) according to the validated protocol used in our diagnostics lab [2]. All the 25 BWS patients did not exhibit any altered methylation pattern in the disease-associated loci *H19* TSS DMR and *KCNQ1T* TSS DMR.

*Methylation-Specific Multiplex Ligation-Dependent Probe* *Amplification*

Twenty of the twenty five BWS patients were analyzed using methylation-specific multiplex ligation-dependent probe amplification (SALSA MLPA ME030 BWS/RSS probemix; MRC-Holland). For five patients, we did not have enough DNA to preform MS_MLPA: BWS7, BWS9, BWS15, BWS23, and BWS25. According to the standardized diagnostic protocol of the diagnostics lab, a patient is diagnosed with BWS if a minimum of three out of four MLPA probes located in *H19* or *KCQ1OT1* are altered in methylation by more than 10% of the average of the methylation level of the at least three negative controls. All the tested patients were considered as wild type.

Mutation analysis of *CDKN1C*

Mutation analysis of the *CDKN1C* gene was performed for all 25 patients according to the validated diagnostic protocol of the genome diagnostic laboratory of the Amsterdam UMC (location AMC). All coding exons with 20bp flanking regions were amplified using polymerase chain reaction (PCR) and Sanger sequencing. Sequenced regions were screened for nonsense and missense mutations and other possible pathogenic variants using CodonCode Aligner software (CodonCode Corporation).

**MLID-BWS patients**

Aberrant imprinted loci in all MLID-BWS were analyzed by Southern blotting hybridization,—combined bisulfite restriction analysis (COBRA) and methylation-specific PCR (MS-PCR)—and described earlier by Bliek et al. (2008) [3]. We additionally investigated MLID in these patients using MS_MLPA (Salsa Mlpa Me034 Multi-locus Imprinting probemix).

**Additional inspection of primary BWS associated loci at 11p15.5**

In addition, we performed a detailed examination of the *H19* TSS DMR in BWS22, BWS24, and BWS25. We checked the overlapping area of significantly hypermethylated CpG sites with MS-MLPA probes. With the knowledge that CTCF binding sites at *H19* TSS DMR may have an effect on BWS phenotype development [4], we checked for disturbed DMPs located in these regions in BWS22, BWS24, and BWS25. Chromosomal location of CTCF-binding sites were filtered out from UCSC and Ensemble (BioMart). The CTCF-binding sites filtering criteria were as follows:

UCSC (Hg19)

- CTCF binding sites by chip-seq from ENCODE/University of Washington
- genomic coordinates: chr11: 2016513 - 2024740
- tissue: blood
- karyotype: normal
- cell lines: GM12878

Ensemble (Hg19)

- BioMart; human regulatory features
- genomic coordinates: chr11: 2016513–2024740
- tissue: peripheral blood
- Feature type: CTCF binding site
- Epigenome name: B cells (PB) Roadmap, GM12878, Monocytes-CD14+ (PB) Roadmap, Natural Killer cells (PB).

We did not detect an overlap between significant DMPs and *H19* MS_MLPA probes in patient BWS22, which explains why the molecular diagnoses was not confirmed.

In BWS24, we saw two significant DMPs overlapping with two out of four *H19* MLPA probes. The difference in methylation in both probes was lower than 10% and did not meet the criteria for confirming the diagnosis using MS_MLPA.

In BWS25, we saw overlap between three significant DMPs with two *H19* MS_MLPA probes and one significant DMP with *KCNQ1OT*1 MS_MLPA probes. However, we did not have enough DNA to carry out the MS-MLPA analysis.

A majority of the significant DMPs located in *H19* TSS DMR in all three patients overlapped the CTCF binding sites and the CpG Island located in this locus, conferring that this locus plays an important regulatory function in the genome. It would appear that even small aberrations of the methylation status in this locus may affect the development of phenotype.

The visualization of this additional inspection is shown in figures 5 and 6.


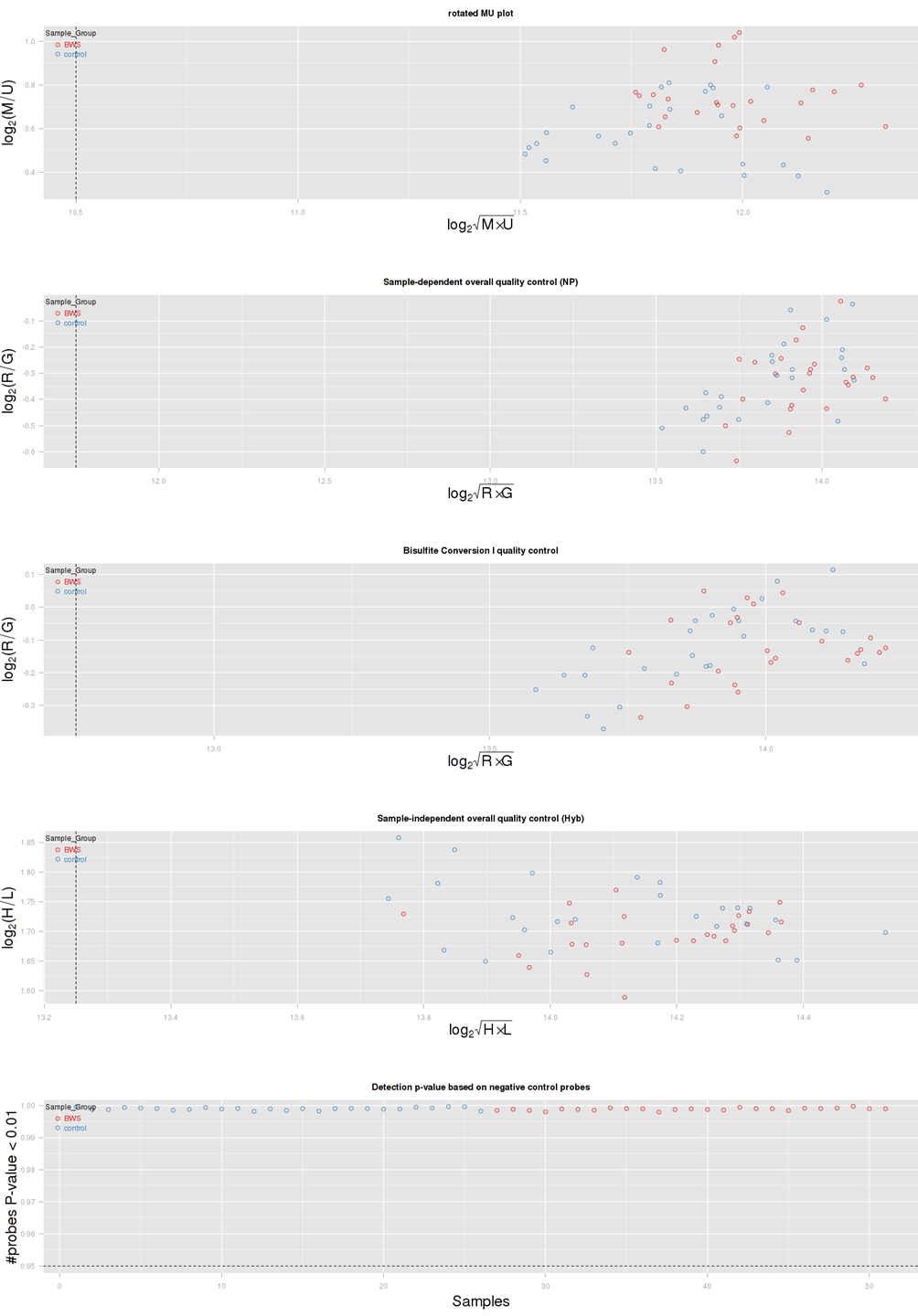


Figure S1. Visual quality control of raw HumanMetylation450k data (MethylAid).


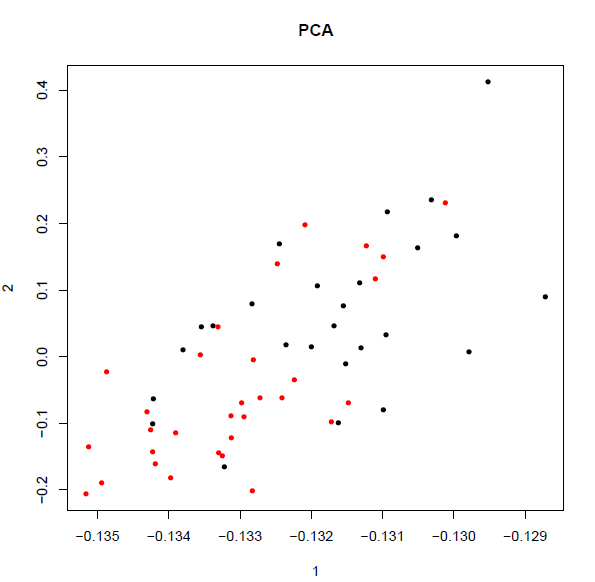


Figure S2. Second principal component analysis. Red dots represent patients and black dots represent controls.

| A | B |
| --- | --- |
|  |  |
| Figure S3. Density plots of betas of MLID-BWS1 patient. A: raw betas; B: normalized betas. | |
|  | |

DNA isolation

Bisulfite treatment

Sample QC

Bisulfite QC

Hybridization

Data filtering (adj.p.value_M<0.05)

Data QC

Data QC

Data pre-processing

**GenomeScan**

Figure S4. Workflow of the single-case analysis.


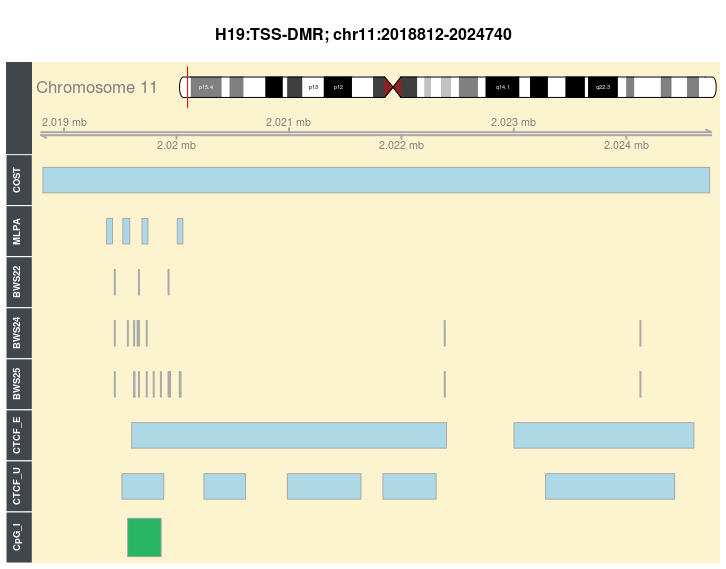


Figure S5. The visualization of *H19* TSS DMR. The COST track represents the genomic localization of the *H19* TSS DMR published by the European Network for Human Congenital Imprinting Disorder; MLPA: genomic localization of *H19* MS-MLPA probes; BWS22, BWS24, and BWS25: hypermethylated DMPs in these three patients, CTCF_E: CTCF-binding sites from Ensemble (BioMart); CTCF_U: CTCF-binding sites from UCSC; CpG_I: CpG island.


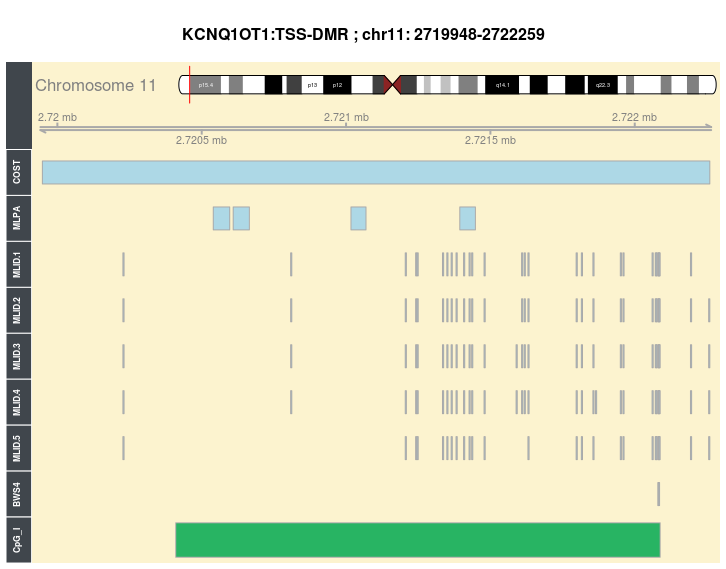


Figure S6. The visualization of *KCNQ1OT*1 TSS DMR. The COST track represents the genomic localization of the *KCNQ1OT1* TSS DMR published by the European Network for Human Congenital Imprinting Disorder; MLPA: genomic localization of *KCNQ1OT1* MS-MLPA probes, MLID.1, MLID.2, MLID.3, MLID. 4, and MLID-BWS5: hypermethylated DMPs in patients MLID-BWS1, MLID-BWS2, MLID-BWS3, MLID-BWS4, and MLID-BWS5; BWS: hypermethylated DMPs in patient BWS5, CpG_I: CpG island.

1. Rezwan FI, Docherty LE, Poole RL, Lockett GA, Arshad SH, Holloway JW, Temple IK, Mackay DJ: **A statistical method for single sample analysis of HumanMethylation450 array data: Genome-wide methylation analysis of patients with imprinting disorders**. *Clin Epigenetics* 2015, **7**:48.

2. Alders M, Bliek J, vd Lip K, vd Bogaard R, Mannens M: **Determination of KCNQ1OT1 and H19 methylation levels in BWS and SRS patients using methylation-sensitive high-resolution melting analysis**. *Eur J Hum Genet* 2009, **17**(4):467–473.

3. Bliek J, Verde G, Callaway J, Maas SM, De Crescenzo A, Sparago A, Cerrato F, Russo S, Ferraiuolo S, Rinaldi MM *et al*: **Hypomethylation at multiple maternally methylated imprinted regions including PLAGL1 and GNAS loci in Beckwith-Wiedemann syndrome**. *Eur J Hum Genet* 2009, **17**(5):611–619.

4. Nativio R, Sparago A, Ito Y, Weksberg R, Riccio A, Murrell A: **Disruption of genomic neighbourhood at the imprinted IGF2-H19 locus in Beckwith-Wiedemann syndrome and Silver-Russell syndrome**. *Hum Mol Genet* 2011, **20**(7):1363–1374.
