# Supplementary material for: Genome-wide methylation profiling of Beckwith-Wiedemann syndrome patients without molecular confirmation after routine diagnostics
Source: Clin Epigenetics. 2019 Mar 21;11:53. doi: 10.1186/s13148-019-0649-6 (PMC6429826; doi:10.1186/s13148-019-0649-6)
Supplement: Supplementary file 6 — Table S5. The summary of identified aberrant CpGs within COST regions. (DOCX 25 kb) [file 13148_2019_649_MOESM6_ESM.docx]

| Table S5. Aberrant methylated COST regions. | | | | | | | |  | |  |  | |  |
| --- | --- | --- | --- | --- | --- | --- | --- | --- | --- | --- | --- | --- | --- |
| **Hypomethylated** | | | | | | | |  |  |  |  |  |  |
|  | BWS4 | | BWS5 | | BWS7 | | Number of patients |  |  |  |  |  |  |
| Gene name | Significant  DMPs | Betadif.  min/max  (mean) | Significant  DMPs | Betadif.  min/max  (mean) | Significant DMPs | Betadif.  min/max  (mean) |  |  |  |  |  |  |  |
| IGF1R |  |  |  |  | 3 | -0.27/-0.18  (-0.22) | 1 |  |  |  |  |  |  |
| NHP2L1 |  |  |  |  | 6 | -0.23/-0.20  (-0.22) | 1 |  |  |  |  |  |  |
| PEG10 |  |  | 2 | -0.22/-0.125  (-0.17) |  |  | 1 |  |  |  |  |  |  |
| L3MBTL1 |  |  |  |  | 24 | -0.34/-0.09  (-0.22) | 1 |  |  |  |  |  |  |
| KCNQ1OT1 | 2 | -0.45/-0.37  (-0.41) |  |  |  |  | 1 |  |  |  |  |  |  |
| **Hypermethylated** | | | | | | | |  |  |  |  |  |  |
|  | BWS7 | | BWS9 | | BWS22 | | BWS24 | | BWS25 | | Number  of  patients |  |  |
| Gene name | Significant  DMPs | Betadif.  min/max  (mean) | Significant DMPs | Betadif.  min/max (mean) | Significant DMPs | Betadif.  min/max (mean) | Significant  DMPs | Betadif.  min/max  (mean) | Significant DMPs | Betadif.  min/max  (mean) |  |  |  |
| H19 |  |  |  |  | 3 | 0.07/0.12  (0.09) | 8 | 0.05/0.14  (0.08) | 26 | 0.06/0.14  (0.10) | 3 |  |  |
| KCNQ1OT1 |  |  | 2 | 0.14/0.46  (0.30) |  |  | 6 | 0.06/0.12  (0.09) | 7 | 0.06/0.13  (0.09) | 3 |  |  |
| MEST |  |  |  |  | 2 | 0.08/0.15  (0.11) | 6 | 0.09/0.14  (0.11) | 8 | 0.08/0.14  (0.11) | 3 |  |  |
| PEG13 |  |  |  |  | 2 | 0.13/0.16  (0.15) | 2 | 0.11/0.12  (0.11) | 3 | 0.09/0.14  (0.12) | 3 |  |  |
| PEG10 |  |  |  |  |  |  | 8 | 0.06/0.11  (0.10) | 7 | 0.07/0.10  (0.09) | 2 |  |  |
| FAM50B |  |  |  |  |  |  | 2 | 0.07/0.10  (0.09) | 8 | 0.08/0.12  (0.10) | 2 |  |  |
| GNAS-A/B |  |  |  |  |  |  | 7 | 0.05/0.13  (0.08) | 4 | 0.06/0.15  (0.10) | 2 |  |  |
| NAP1L5 |  |  |  |  |  |  | 3 | 0.08/0.22  (0.13) | 2 | 0.07/0.10  (0.10) | 2 |  |  |
| ZNF331  variant_7 |  |  |  |  |  |  | 2 | 0.11/0.11  (0.11) | 3 | 0.10/0.15  (0.12) | 2 |  |  |
| ZNF331  variants_4-6 |  |  |  |  |  |  | 2 | 0.09/0.09  (0.09) | 3 | 0.08/0.12  (0.10) | 2 |  |  |
| MCTS2P |  |  |  |  |  |  | 2 | 0.06/0.10  (0.08) | 3 | 0.08/0.12  (0.10) | 2 |  |  |
| GNAS-NESP |  |  |  |  |  |  | 2 | 0.11/0.13(  0.12) | 3 | 0.10/0.13  (0.11) | 2 |  |  |
| DIRAS3 |  |  |  |  |  |  | 2 | 0.06/0.12  (0.09) | 3 | 0.07/0.09  (0.08) | 2 |  |  |
| SNRPN/SNURF |  |  |  |  |  |  | 2 | 0.11/0.12  (0.11) | 2 | 0.11/0.11  (0.11) | 2 |  |  |
| MEG3 |  |  |  |  |  |  | 5 | 0.07/0.11  (0.09) | 6 | 0.06/0.10  (0.09) | 2 |  |  |
| ZNF597 |  |  |  |  |  |  | 2 | 0.12/0.14  (0.13) | 2 | 0.09/0.13  (0.11) | 2 |  |  |
| IGF2_3'UTR |  |  |  |  |  |  | 3 | 0.11/0.20  (0.15) |  |  | 1 |  |  |
| PEG3 |  |  |  |  |  |  |  |  | 2 | 0.07/0.10  (0.09) | 1 |  |  |
| NHP2L1 |  |  |  |  |  |  |  |  | 2 | 0.09/0.10  (0.09) | 1 |  |  |
| L3MBTL1 |  |  |  |  |  |  | 2 | 0.09/0.09  (0.09) |  |  | 1 |  |  |
| GNAS-AS1 |  |  |  |  |  |  |  |  | 2 | 0.08/0.09  (0.09) | 1 |  |  |
| ZDBF2 | 4 | 0.14/0.30  (0.24) |  |  |  |  |  |  |  |  | 1 |  |  |
| INPP5F |  |  |  |  |  |  |  |  | 3 | 0.18/0.28  (0.25) | 1 |  |  |
| NNAT |  |  |  |  |  |  | 3 | 0.08/0.15  (0.10) |  |  | 1 |  |  |
